# Preparation of rapeseed oil with superhigh canolol content and superior quality characteristics by steam explosion pretreatment technology

**DOI:** 10.1002/fsn3.1502

**Published:** 2020-03-31

**Authors:** Gaiwen Yu, Tingting Guo, Qingde Huang

**Affiliations:** ^1^ Oil Crops Research Institute Chinese Academy of Agricultural Sciences Wuhan China; ^2^ Hubei Key Laboratory of Lipid Chemistry and Nutrition Wuhan China; ^3^ Oil Crops and Lipids Process Technology National & Local Joint Engineering Laboratory Wuhan China

**Keywords:** canolol, high‐temperature roasting, rapeseed oil, sinapic acid, Steam explosion pretreatment

## Abstract

In this study, rapeseed was pretreated by steam explosion pretreatment technology and subsequently pressed to prepare rapeseed oil. GC, UPLC, and HPLC techniques were employed to analyze the quality characteristics of the rapeseed oil, including the canolol content and other quality characteristics. Additionally, the effect of steam explosion pretreatment technology on the canolol content of rapeseed oil was studied and the formation mechanism of canolol elucidated. The results revealed that when the steam explosion pressure reached 1.0 MPa, the canolol content of the tested oil increased from 41.21 to 2,168.69 mg/kg (52.63‐fold increase) and that sinapic acid played a significant role in the conversion of canolol. Thus, the sinapine was converted into the intermediate (sinapic acid) by hydrolysis, which in turn was transformed into canolol through decarboxylation. The instantaneous high‐energy environment generated by steam explosion pretreatment could intensify the hydrolysis and decarboxylation reactions of sinapine and sinapinic acid, thereby significantly increasing the canolol content of the oil. To prove the superiority of steam explosion pretreatment, we compared it with other pretreatment technologies, including traditional high‐temperature roasting and popular microwave pretreatment. The results revealed that rapeseed oil prepared by steam explosion pretreatment displayed the best quality characteristics. This study can be a reference for the preparation process of rapeseed oil with superhigh canolol content and superior quality characteristics using steam explosion pretreatment.

## Graphical abstract

The objective of the present study was to carry out steam explosion experiments on rapeseed, analyze the formation mechanism of Canolol, and compare the lipid characteristics (physicochemical parameters, fatty acid composition, phytochemicals (tocopherols, phytosterols, polyphenols), and in vitro antioxidant activities).

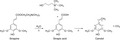

## 1 INTRODUCTION

Rapeseed is rich in polyphenols, with contents 10–30 times higher than those of other oil seeds (Yu, Bals, Grimi, & Vorobiev, 2015). Canolol is one of the most important rapeseed polyphenols, with many beneficial characteristics, including strong antioxidant, anticancer, and lipid‐lowering effects; it is also effective in the prevention and treatment of cardiovascular diseases (Kraljić, Brkan, Škevin, Srček, & Radošević, 2019; Tan, Mailer, Blanchard, Samson, & Agboola, 2011; Xia et al., 2018). Therefore, this polyphenol is a potentially important natural antioxidant that can be applied to healthcare products (Han et al., 2017). Unfortunately, although polyphenols are abundant in rapeseed, most remain in the rapeseed cake after oil production and only a small amount of polyphenols is transferred to the rapeseed oil (Zheng, Yang, Zhang, & Huang, 2017).

There are significant differences in the canolol contents and other quality characteristics of rapeseed oils, according to the different pretreatment technologies adopted (Siger, Gawrysiak‐Witulska, & Bartkowiak‐Broda, 2017; Siger & Michalak, 2016; Zheng, Yang, Zhou, Liu, & Huang, 2015). Siger et al. (2017) investigated the influence of different roasting conditions on the quality characteristics of rapeseed oil, including the canolol, tocopherol, and plastochromanol‐8 contents. They reported 90‐ and 46‐fold increases in canolol in two varieties, namely the PN1 03/1i/14 and PN1 563/1i/14 lines, respectively. Moreover, when roasting the PN1 03/1i/14 line at 180°C for 15 min, the tocopherol content of the tested rapeseed oil increased by 17%–18%, while the plastochromanol‐8 content increased approximately twofold. Wroniak et al. (2016) investigated the effects of the moisture contents (7 and 9%) and microwave radiation (800 W for 0, 3, and 7 min) on the microstructure and quality characteristics of two rapeseed varieties (Kana and Bakara). The results indicated that the tocopherol amounts and oxidative stabilities were significantly affected by the different microwave pretreatment conditions. Moreover, the canolol contents increased to 926.42 and 821.86 μg/g (56.69‐ and 48.43‐fold increases), respectively, under 800 W microwave radiation for 7 min at 7% moisture level, while the fatty acid compositions were not significantly affected (*p* > .05).

To date, there are relatively few studies on the influence of steam explosion pretreatment technology on the canolol content and other quality characteristics of rapeseed oil. Steam explosion technology is a physicochemical pretreatment method that transforms thermal energy into mechanical energy and achieves the separation and structural changes of macromolecule substances in components through instantaneous pressure release and expansion under a high‐temperature and pressure environment (Li et al., 2019; Shi, Li, Li, Cheng, & Zhu, 2019; Zhang et al., 2019). Consequently, this research performed steam explosion pretreatment experiments on rapeseed to analyze the formation mechanism of canolol and evaluate the quality characteristics, including the physicochemical properties, fatty acid profiles, phytochemical (tocopherol, phytosterol, and polyphenol) contents, and in vitro antioxidant activities. The findings of this research can serve as guidance for the industrial preparation of rapeseed oil of superhigh canolol content and are also of important reference significance for the development of processes that afford superior quality rapeseed oil.

## 2 MATERIALS AND METHODS

### 2.1 Experimental materials and pretreatment

Double low rapeseed (Brassicanapus) samples were collected from Wuhan Zhongyou Grand Science and Technology Industry Co., Ltd.

Steam explosion pretreatment was carried out on an XSS–QPD multifunctional air expander (Wuhan KINHE Food Machinery Co.,Ltd.). A certain amount of rapeseed was weighed, the moisture of rapeseed was adjusted to about 12%, when the water quality was balanced, rapeseed was put into the air puffing bin, and the heating temperature by constant temperature system was controlled (about 220°C). When the pressure of the silo reached the set pressure (0.4 MPa, 0.6 MPa, 0.8 MPa, 1.0 MPa, and 1.2 MPa), it was within a very short time (0. 0875s) to complete the pressure release to realize steam explosion (Li et al., 2019).

A sealed microwave digestion instrument (maximum power, 4,800 W and frequency, 2,450 MHz; CEM Corporation) was employed to simulate the most common microwave pretreatment process currently in use. Some fresh rapeseed was accurately weighed, and the fresh rapeseed moisture was adjusted to 12%, when balanced, the rapeseed was pretreated under 800 W for 7 min at a frequency of 2,450 MHz and then cooled to room temperature.

Referring to the documentation (Siger et al., 2017), traditional high‐temperature roasting pretreatment was performed on a GSCH‐series multi‐function heat‐roasting machine (Henan Ruiguang Machinery Co., Ltd., China); some rapeseed were weighed; then, the temperature of the multi‐function heat‐roasting machine was set through the temperature control function; a short period of time (about 1 min) was stabilized after the roasting machine was heated to 180°C; and finally, the weighing rapeseed was roasted for 15 min, and then, it was cooled naturally to room temperature.

### 2.2 Preparation of rapeseed oil

In view of the loss of water in rapeseed under different pretreatment conditions, the moisture content of rapeseed was controlled by adding distilled water to between 6% and 7%, then squeezed; at the same time, the pressing temperature was controlled within 65°C, and the oil phase after filtering was pressed rapeseed oil. What's more, rapeseed oil was weighed to calculate the oil yield.

### 2.3 Determinations

#### 2.3.1 Oil yield contents

The oil yield contents were determined according to ISO659.2009, using analytical grade petroleum for gravimetric analysis in Soxhlet apparatus (B–811, Buchi Labortechnik AG) for 8 hr.

The oil yield content was calculated as:

$$Y=\frac{M_{1}}{M_{2}\times X}\times 100\%$$

where Y is the oil yield content (%), M_1_ and M_2_ are the rapeseed oil and rapeseed masses, respectively (g), and X is the rapeseed oil content (%).

#### 2.3.2 Physicochemical properties

The physicochemical properties [acid (AV) and peroxide (POV) values] of the tested rapeseed oil samples were measured according to the AOCS official methods  (Firestone, 2003). 

#### 2.3.3 Canolol and other phenolic compounds

The contents of canolol and other phenolic compounds were determined according to a previously reported method. The analysis was performed on an Acquity Ultraperformance liquid chromatograph (UPLC; Waters) combined with a photodiode array (PDA) detector and the Waters Acquity BEH Shield RP18 (100 × 2.1 mm, 1.7 μm, Waters). The injection volume was 3 μl, and the temperature of the column was 30°C. The extracts were eluted with 2% acetic acid (w/w; mobile phase A) and 100% methanol (mobile phase B) at a flow of 0.21 ml/min. The gradient elution was as follows: 5%–25% B (7.40 min), 25%–29% B (2.67 min), 29%–36% B (6.66 min), 36%–45% B (6.67 min), 45%–65% B (2 min), 45%–65% B (2 min), 65%–5% B (2 min), and 5% B (2.67 min). The quantitative determination of the phenolic compounds was implemented on a UPLC–PDA detector at the detection wavelengths 270 nm for canolol and 330 nm for the other compounds.

#### 2.3.4 Phytosterols

The phytosterol contents were determined as follows: 10 ml of 2 mol/L KOH in ethanol was used to saponify 0.2 g (accuracy 0.0001 g) of the tested oil samples and 0.5 ml of 0.5 mg/ml 5*α‐*cholestane (internal standard) at 60°C for 60 min; the unsaponifiable compositions were obtained with hexane. The hexane layer was dried over anhydrous sodium sulfate and silylated using 100 µl *N*, O–bis (trimethylsilyl) trifluoroacetamide + 1% trimethylchlorosilane (BSTFA + TMCS) at 105°C for 15 min. The mixture was then dissolved in 1 ml hexane for further analysis on an Agilent 6890A gas chromatography system (Agilent) equipped with a DB–5HT column (30 m × 0.32 mm, 0.1 μm; Agilent). The nitrogen (carrier gas) flow rate was 2.0 ml/min, while the detector and injection temperatures were maintained at 320°C. The oven temperature was programmed as follows: an original temperature of 60°C for 1 min, increased to 310°C at 4°C /min and maintained at this temperature for 10 min. The split ratio was 25:1, and the injection volume was 1 μl.

#### 2.3.5 Tocopherols

The tocopherol contents in rapeseed oil were quantified using the AOCS Official Method Ce 8–89 with slight modifications. Briefly, 2 g (accuracy 0.0001 g) tested oil sample was weighed in a 25 ml volumetric flask, dissolved in a hexane layer, and then filtered through a 0.22 μm polytetrafluoroethylene filter. The treated samples (20 μl) were measured by high‐performance liquid chromatography (HPLC; LC–20A, Shimadzu Corp.) on an SIL100A column (250 × 4.6 mm, 5 μm; GL Sciences Inc.). The flow rate of the mobile phase, which comprised a hexane–isopropanol mixture (99.5:0.5, v/v), was 1 ml/min, and the α‐ and γ‐tocopherol contents were analyzed at 292 and 298 nm, respectively.

#### 2.3.6 Fatty acids

The rapeseed oil samples were methylated with sodium methoxide and subsequently analyzed using an Agilent 7890A gas chromatography system (Agilent) equipped with an HP–INNOWAX capillary column (30 m × 0.32 mm, 0.25 μm; Agilent Corp.). The injector and detector temperatures were maintained at 250°C. The flow rate of the N_2_ (carrier gas), with an 80:1 split ratio, was set to 1.5 ml/min. The oven temperature was programmed as follows: an original temperature of 210°C for 9 min, increased to 230°C at 20°C /min and maintained at this temperature for 10 min. The injection volume in this experiment was 1 μl. The analysis of the fatty acid composition in the treated oil samples was achieved by comparing the afforded retention times with those of standard fatty acids.

#### 2.3.7 Antioxidant capacity assays

Briefly, exactly 1.25 g rapeseed oil was placed in a 10 ml centrifuge tube. About 1.5 ml 80% methanol aqueous solution was then added with shaking (HA9–HMV multi‐vortex mixer; Wuxi Voshin Instruments Manufacturing Co., Ltd.), at 4,863 g, for 5 min in the dark. The supernatant was carefully transferred into another tube, and the extract was separated with 1.5 ml 80% methanol aqueous solution. This process was repeated three times.

##### 2,2—Diphenyl‐1‐picrylhydrazyl (DPPH) method

0.5 ml extract was added to 2.5 ml of DPPH in methanol (0.0964 mol/L). The mixture was shaken vigorously for 30 s and subsequently allowed to react at room temperature (about 25°C), in the dark, for 30 min. The absorbance was analyzed for pure methanol (blank) at 515 nm using a DU 800 UV/Visible Spectrophotometer (Beckman Coulter Inc.). The DPPH free radical scavenging assay was obtained as ([A control–A sample]/A control) ×100%. Trolox reagent was used as the standard for the calibration curve (DPPH = 5.8329 × C_Trolox_ +0.01; *R*_2_ = .9994). The results were stated as micromolar Trolox equivalents per 100 g oil (μmol TE/100 g).

##### Fluorescence recovery after photobleaching (FRAP) method

A freshly prepared FRAP working solution (2.5 ml 10 mmol/L tripyridyl triazine (TPTZ) solution in 40 mmol/L HCl, 2.5 ml 20 mmol/L FeCl_3_, and 25 ml 0.1 mol/L acetate buffer; pH 3.6) was hatched at 37°C for 10 min. Next, 0.5 ml extract and 2 ml FRAP working solution were added to a 10 ml volumetric flask containing redistilled water. The mixture was maintained in the dark at 25°C for 20 min. The reagent blank was analyzed at 593 nm using a TU‐1901 Dual Beam UV‐vis spectrophotometer (Beijing Purkinje General Instrument Co., Ltd.). Trolox reagent was used as the standard for the calibration curve (FRAP = 0.0088 × C_Trolox_−0.0179; *R*_2_ = .995). The results were stated as micromolar Trolox equivalents per 100 g oil (μmol TE/100 g).

#### 2.3.8 Oxidation induction time (OIT)

According to the AOCS Cd 12b–92 method, the OITs of the rapeseed oil samples were determined on a Metrohm Rancimat 743 (Herisau). A 3 g sample was weighed and placed into a glass reaction tube, heated at 110°C, and then passed through 20 L/hr of dried and cleaned air. The volatile organic acid was collected in a measuring vessel comprising 50 ml distilled water. As the oxidation reaction progressed, the water conductivity was automatically measured and the results were reported in hours (hr).

#### 2.3.9 Data analyses

All the analyses were implemented in triplicate and presented as the means ± standard errors. Statistical analyses were performed using the SPSS program (SPSS 23.0 for Windows, SPSS Inc.). Duncan's test at the 5% significance level (*p* < .05) and one‐way analysis of variance (ANOVA) were used to determine the significance differences.

## 3 RESULTS AND DISCUSSIONS

### 3.1 Effects of steam explosion pretreatment technology on the canolol content of rapeseed oil

**Figure 1:**
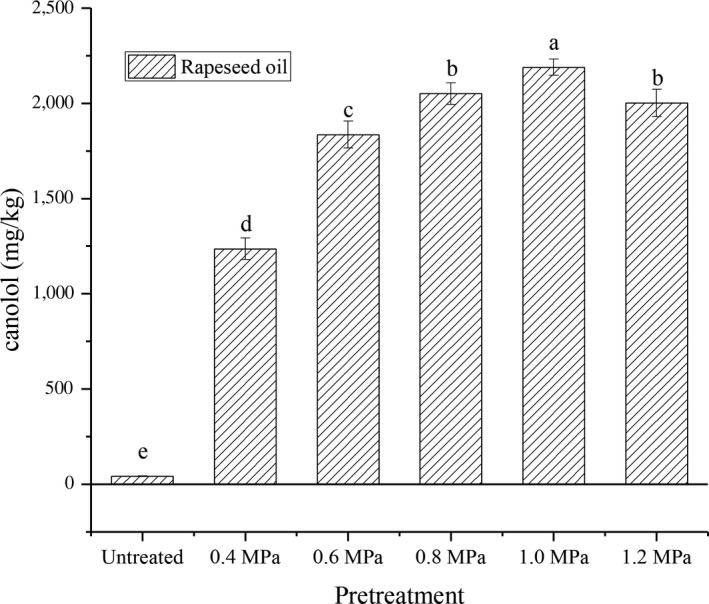
Effect of the different steam explosion pressures on the canolol content in rapeseed oil

The influence of the steam explosion pressure on the canolol content of the tested rapeseed oil was illustrated in Figure 1. The results revealed that the canolol content increased with the extension of the explosion pressure range from 0.4 to 1.0 MPa (*p* < .05), where it reached a maximum (2,168.69 mg/kg). However, when the steam explosion pressure was further extended to 1.2 MPa, a decrease in the canolol content was observed. It was considered that sinapic acid and its derivatives were directly or indirectly pyrolyzed to canolol. However, canolol underwent thermal degradation and became combined with the protein with the extension of the explosion pressure to 1.2 MPa, thereby resulting in its partial decomposition (Wroniak et al., 2016; Yang et al., 2014). This was consistent with the results reported by Spielmeyer, who observed that the temperature had a significant effect on the canolol content of the tested rapeseed oil (Spielmeyer, Wagner, & Jahreis, 2009).

### 3.2 Effects of three different pretreatment technologies on the canolol content of rapeseed, rapeseed oil, and rapeseed cake

The influences of different pretreatment technologies on the canolol content of rapeseed, rapeseed oil, and rapeseed cake were compared in Table 1. The results revealed that after steam explosion pretreatment, the canolol in rapeseed increased from 18.16 to 185.26 mg/kg, while that in rapeseed oil increased from 41.21 to 2,168.69 mg/kg (52.63‐fold increase). Compared with the traditional high‐temperature roasting popular and microwave pretreatment, the canolol content of the tested rapeseed oil increased by 124.36 and 112.30%, respectively. Moreover, the residual rates of canolol in the rapeseed cakes from the different pretreatment methods (untreated, roasted, microwave and steam explosion pretreatment) were, respectively, 67.4, 59.6, 58.3, and 48.0%. These results indicated that cytoplasm aggregation was more concentrated and the cell liquid overflow was more beneficial with steam explosion pretreatment (Zago et al., 2015). This was reflected by the lowest residual canolol value observed in the steam‐exploded rapeseed cake. Yang et al. (2014) pretreated rapeseed with microwave technology under 800 W at different times followed by pressing to prepare the oil samples. The results manifested that microwave treatment dramatically affected the distribution and content of phenolic compounds in the rapeseeds and oil from them, and the formation of canolol was dramatically correlated with the loss of sinapic acid and sinapine (*r* = .997 and *r* = .952, respectively); furthermore, the canolol content increased to a maximum (sixfold increase) under 800 W for 7 min.

**Table 1:** Effects of three different pretreatment technologies on the canolol content of rapeseed, rapeseed oil, and rapeseed cake

Pretreatment	Untreated	Roasted	Microwave	Steam explosion
Rapeseed	18.16 ± 1.58^c^	85.50 ± 4.06^b^	91.36 ± 4.82^b^	185.26 ± 5.96^a^
Rapeseed oil	41.21 ± 3.12^c^	953.22 ± 56.45^b^	1,021.53 ± 65.48^b^	2,168.69 ± 88.95^a^
Rapeseed cake	12.24 ± 1.06^c^	49.13 ± 3.28^b^	53.58 ± 3.74^b^	89.04 ± 4.54^a^

Note

Different letters in a row mean significant difference at the 5% level.

Roasted, microwave, steam explosion pretreatment methods, respectively, represent 180°C for 15 min, 800 W for 6 min at a frequency of 2,450 MHz and steam explosion pressure at 1.0 MPa.

### 3.3 Effects of three different pretreatments on the contents of various phenolic compounds in rapeseed and analysis of the formation mechanism of canolol

Table 2 compared the influence of steam explosion, microwave, and roasting pretreatment technologies on the contents of various phenolic compounds in rapeseed. The results revealed that the contents of six of the rapeseed polyphenols decreased after pretreatment. Particularly, the sinapic acid (SA), sinapolyl glucoside (SG), sinapoylcholine thiocyanate glucoside (SPTG), sinapoylmalic acid (SM), and disinapoyl gentiobioside (DSGG) contents decreased significantly, while only a slight notably, for all seven phenolic compounds, the most significant changes were observed in the steam‐explosion‐pretreated sample.

**Table 2:** Effects of three different pretreatments on the contents of various phenolic compounds in rapeseed (mg/kg)

Pretreatment	Untreated	Roasted	Microwave	Steam explosion
SP	275.51 ± 6.68^a^	258.24 ± 7.62^b^	256.39 ± 6.08^b^	243.42 ± 5.95^b^
SA	41.34 ± 3.12^a^	29.16 ± 2.06^b^	18.95 ± 1.62^c^	4.02 ± 0.44^b^
SG	75.25 ± 2.94^a^	43.69 ± 3.52^b^	18.24 ± 1.82^c^	3.96 ± 0.36^d^
SPTG	46.14 ± 2.45^a^	37.18 ± 3.84^b^	28.39 ± 3.02^c^	16.25 ± 1.14^d^
SM	65.41 ± 3.21^a^	52.39 ± 2.25^a^	37.42 ± 2.16^b^	7.26 ± 1.27^a^
DSGG	84.64 ± 6.88^a^	62.19 ± 4.98^b^	24.26 ± 2.96^c^	9.21 ± 0.88^d^
Canolol	18.16 ± 1.75^c^	86.50 ± 4.82^b^	90.93 ± 5.97^b^	185.26 ± 8.04^a^

Note

Different letters in a row mean significant difference at the 5% level.

Roasted, microwave, steam explosion pretreatment methods, respectively, represented 180°C for 15 min, 800 W for 6 min at a frequency of 2,450 MHz and steam explosion pressure at 1.0 MPa.

SP, SA, SG, SPTG, SM, and DSGG, respectively, represented sinapine, sinapic acid, sinapoyl glucoside, sinapoylcholine thiocyanate glucoside, sinapoylmalic acid, and disinapoyl gentiobioside.

**Figure 2:**
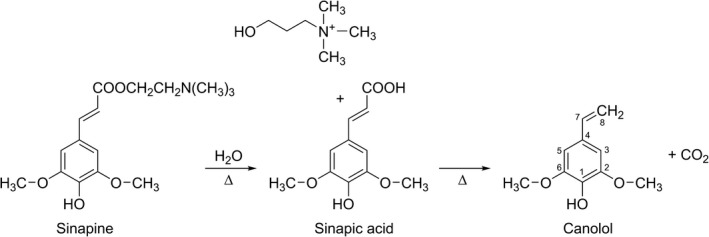
Hydrolysis and decarboxylation reaction of sinapine and sinapic acid to form canolol

The reaction route of canolol formation from the hydrolysis and decarboxylation of SP and SA was demonstrated in Figure 2. It was concluded that SP was hydrolyzed to produce SA, which was then transformed into canolol via decarboxylation (Erika et al., 2015). Sinapic acid, as an intermediate product of canolol formation, plays an important role in the transformation of canolol. Up to now, there have been basically no reports on the mechanism of the canolol production by other sinapic acid derivatives (SG, SPTG, SM, and DSGG), and further research is being made in our laboratory. Cong et al. (2019) studied the influence of the popular microwave pretreatment on the distribution of SA and its derivatives in rapeseed, and found that microwave pretreatment technology affected the distributions of SA and its derivatives, which contributed to the formation of canolol in rapeseed. Analytical results have suggested that steam explosion pretreatment followed by an instantaneous high‐energy environment could accelerate the breaking and recombination of the reaction bonds and intensify the hydrolysis and decarboxylation of SP and SA (Seçmeler, Üstündağ, Fernández‐Bolaños 2018; Leskinen et al., 2017; Lopezlinares et al., 2015). These results well agreed with the results in this study, where compared with the other pretreatment methods, steam explosion pretreatment afforded rapeseed oil with the highest canolol content.

### 3.4 Effects of three different pretreatments on some quality characteristics of rapeseed oil

Table 3 compared different quality characteristics of rapeseed oil samples prepared by different pretreatment technologies. The results revealed that the different pretreatments afforded significantly different quality characteristics (*p* < .05). Thus, after steam explosion pretreatment, the oil yield of the tested rapeseed oil increased from 69.26% to 80.11%, a value that was significantly higher than those observed for the roasted‐ (74.77%) and microwave‐ (75.47%) pretreated samples. This superior yield was attributed to steam expansion during the pressure release process, which led to more thorough destruction of the oil cells and thus a maximized oil yield (Niu et al., 2015).

**Table 3:** Effects of three different pretreatments on some quality characteristics of rapeseed oil

Pretreatment	Untreated	Roasted	Microwave	Steam explosion
Oil yield (%)	69.26 ± 2.83^c^	74.77 ± 2.01^b^	75.47 ± 1.98^b^	80.11 ± 2.79^a^
AV (mg/g oil)	1.15 ± 0.01^b^	1.56 ± 0.00^a^	1.45 ± 0.22^a^	1.43 ± 0.12^a^
POV (meq. O_2_/kg oil)	0.65 ± 0.07^c^	0.75 ± 0.03^ab^	0.78 ± 0.02^a^	0.72 ± 0.01^ab^
Phytosterols (mg/kg oil)	6,008.49 ± 90.51^c^	6,245.76 ± 98.99^bc^	6,359.28 ± 113.14^ab^	6,622.65 ± 127.28^a^
Tocopherols (mg/kg oil)	635.24 ± 10.03^c^	654.84 ± 14.28^bc^	678.52 ± 12.25^ab^	686.67 ± 12.84^a^
DPPH (μmol/100 g oil)	16.52 ± 1.18^d^	169.49 ± 2.96^c^	240.46 ± 3.87^b^	270.12 ± 1.64^a^
FRAP (μmol/100 g oil)	20.56 ± 0.78^d^	175.26 ± 5.47^c^	278.81 ± 4.94^b^	305.12 ± 5.84^a^
OIT (hr)	10.62 ± 0.08^d^	15.23 ± 0.32^a^	13.86 ± 0.16^c^	14.58 ± 0.45^b^

Note

Different letters in a row mean significant difference at the 5% level.

AV, POV, DPPH, FRAP, OIT, respectively, represented acid value, peroxide value, DPPH free radical scavenging ability, ferric ion reducing antioxidant power, and oxidation induction time.

Roasted, microwave, steam explosion pretreatment methods, respectively, represented 180 ℃ for 15 min, 800 W for 6 min at a frequency of 2,450 MHz and steam explosion pressure at 1.0 MPa.

The AVs and POVs of the tested rapeseed oil samples slightly increased after all three pretreatment methods, owing to the long oxidation of rapeseed oil under high temperature (Xu et al., 2018). However, although the AV and POV of the steam‐explosion‐pretreated rapeseed oil slightly increased after steam explosion pretreatment, these values were significantly lower than the limit standards. Moreover, these two values were markedly lower than those afforded by traditional high‐temperature roasting and popular microwave pretreatment. This was attributed to the extremely short completion time of the steam explosion pretreatment compared with those of the other two methods.

The influence of the three different pretreatment methods on the micronutrient contents of the respective pressed rapeseed oil samples was also illustrated in Table 3. The results manifested a marked difference in the phytosterol and tocopherol contents of the three tested rapeseed oil samples (*p* < .05). The highest phytosterol and tocopherol contents were observed in the rapeseed oil pretreated with steam explosion technology. These results were attributed to the instantaneous high‐energy environment formed during the steam expansion process to accelerate fat accumulation, which was also more conducive to the dissolution of some fat‐soluble micronutrient components (Liu & Chen, 2015). Thus, when the steam explosion pressure reached 1.0 MPa, the phytosterol and tocopherol contents were, respectively, 6,622.65 and 686.67 mg/kg, which were markedly superior to those afforded by the other two pretreatment methods.

The effects of the three pretreatments on the DPPH, FRAP, and OIT values of pressed rapeseed oil were also investigated (Table 3). The results indicated that all three pretreatment processes significantly increased the oxidation stability of rapeseed oil (*p* < .05). This increase was more significant in the steam‐explosion‐pretreated sample. Thus, when the steam explosion pressure reached 1.0 MPa, the DPPH, FRAP, and OIT values of the tested rapeseed oil sample were 270.12 μmol/100 g, 305.12 μmol/100 g, and 14.58 hr, respectively. Notably, compared with the values observed in the unpretreated samples, the DPPH, FRAP, and OIT values of the steam‐explosion‐pretreated oil increased by 15.35, 13.84, and 0.37 times, respectively, possibly owing to the high levels of polyphenols, particularly of canolol.

### 3.5 Effects of three different pretreatments on the main fatty acid contents in rapeseed oil

The effects of the three different pretreatment methods on the main fatty acid composition of rapeseed oil were compared in Table 4. The results revealed the following fatty acid composition of rapeseed oil prepared by steam explosion pretreatment: palmitic acid (3.83%), stearic acid (2.46%), oleic acid (65.91%), linoleic acid (17.09%), and linolenic acid (7.81%). Notably, the three pretreatment methods had little influence on the fatty acid composition of rapeseed oil (*p* > .05). These results were well in agreement with those reported by Małgorzata, whereby the fatty acid composition of rapeseed oil was not significantly affected by changes of the microwave radiation in their experiments (Małgorzata et al., 2016).

**Table 4:** Effects of different pretreatments on the main fatty acid contents in rapeseed oil (%)

Fatty acid	Untreated	Roasted	Microwave	Steam explosion
Palmitic (C16:0)	3.76 ± 0.03^a^	3.78 ± 0.04^a^	3.78 ± 0.02^a^	3.83 ± 0.03^a^
Stearic (C18:0)	2.46 ± 0.06^a^	2.45 ± 0.08^a^	2.46 ± 0.03^a^	2.46 ± 0.02^a^
Oleic (C18:1)	65.88 ± 0.04^a^	66.02 ± 0.02^a^	66.04 ± 0.10^a^	65.91 ± 0.07^a^
Linoleic (C18:2)	17.04 ± 0.10^a^	17.13 ± 0.10^a^	17.06 ± 0.05^a^	17.09 ± 0.02^a^
Linolenic (C18:3)	7.83 ± 0.17^a^	7.75 ± 0.19^a^	7.72 ± 0.12^a^	7.81 ± 0.10^a^
Eicosanoic (C20:1)	1.84 ± 0.06^a^	1.77 ± 0.07^b^	1.79 ± 0.08^b^	1.79 ± 0.05^b^
Erucic (C22:1)	0.52 ± 0.06^b^	0.56 ± 0.05^b^	0.68 ± 0.06^ab^	0.75 ± 0.04^a^
SFA	6.22 ± 0.00^a^	6.23 ± 0.08^a^	6.24 ± 0.06^a^	6.29 ± 0.06^a^
MUFA	68.26 ± 0.04^a^	38.36 ± 0.15^a^	68.50 ± 0.14^a^	68.47 ± 0.12^a^
PUFA	24.87 ± 0.12^a^	24.88 ± 0.17^a^	24.78 ± 0.16^a^	27.90 ± 0.15^a^
UFA	93.13 ± 0.10^a^	93.24 ± 0.19^a^	93.28 ± 0.18^a^	93.38 ± 0.29^a^

Note

Different superscript letters in a row mean significant difference at the 5% level.

Roasted, microwave, steam explosion pretreatment methods, respectively, represented 180°C for 15 min, 800 W for 6 min at a frequency of 2,450 MHz and steam explosion pressure at 1.0 MPa.

Abbreviations: MUFA, monounsaturated fatty acids; PUFA, polyunsaturated fatty acids; SFA, saturated fatty acids; UFA, unsaturated fatty acids.

## 4 CONCLUSIONS

In this study, three pretreatment methods, namely traditional high‐temperature roasting, popular microwave, and steam explosion pretreatments, were performed to prepare three different rapeseed oil samples. The canolol, polyphenol, phytosterol, and tocopherol contents as well as other quality characteristics of the three samples were then analyzed. The results indicated that steam explosion pretreatment afforded increases in the oil yield content (from 69.26% to 80.11%), AV and POV (slight increase), and phytosterol and tocopherol contents (varying degrees). On the other hand, the pretreatment had almost no effect on the fatty acid composition of the oil. The canolol content in the rapeseed oil was the highest when the steam explosion pressure reached 1.0 MPa, increasing from 41.21 to 2,168.69 mg/kg, this resulted in a 52.63‐fold increase over the unpretreated samples and a 124.36 and 112.30% increase over that of the high‐temperature roasting and microwave pretreated sample.

As an intermediate of canolol, SA played an important role in the formation of canolol. It was concluded that canolol was produced by the conversion of the intermediate product through hydrolysis of SP and subsequent decarboxylation of the intermediate. Moreover, it was considered that steam explosion technology provided an instantaneous high‐energy environment that accelerated the breaking and recombination of the reaction bonds and intensified the hydrolysis and decarboxylation of SP and SA. Therefore, compared with the other pretreatment methods, steam explosion pretreatment afforded rapeseed oil with the highest canolol content. The results suggest that this study can be of reference for the preparation process of rapeseed oil with a superhigh canolol content and superior quality characteristics via steam explosion pretreatment technology.

## CONFLICT OF INTEREST

The authors declared that we had no any conflict of interest.

## ETHICAL APPROVAL

The study did not include any animal or human tests.
